# Sequence-Aware Vision Transformer with Feature Fusion for Fault Diagnosis in Complex Industrial Processes

**DOI:** 10.3390/e27020181

**Published:** 2025-02-08

**Authors:** Zhong Zhang, Ming Xu, Song Wang, Xin Guo, Jinfeng Gao, Aiguo Patrick Hu

**Affiliations:** 1 School of Electrical and Information Engineering, Zhengzhou University, Zhengzhou 450001, China; iezzhang@zzu.edu.cn (Z.Z.); wangsong61@163.com (S.W.); iexguo@zzu.edu.cn (X.G.); jfgaozzu@163.com (J.G.); 2 School of Integrated Circuits, Zhongyuan University of Technology, Zhengzhou 451191, China; 3 Department of Electrical, Computer and Software Engineering, The University of Auckland, Auckland 1010, New Zealand; a.hu@auckland.ac.nz

**Keywords:** fault diagnosis, vision transformer, feature fusion, Tennessee Eastman process

## Abstract

Industrial fault diagnosis faces unique challenges with high-dimensional data, long time-series, and complex couplings, which are characterized by significant information entropy and intricate information dependencies inherent in datasets. Traditional image processing methods are effective for local feature extraction but often miss global temporal patterns, crucial for accurate diagnosis. While deep learning models like Vision Transformer (ViT) capture broader temporal features, they struggle with varying fault causes and time dependencies inherent in industrial data, where adding encoder layers may even hinder performance. This paper proposes a novel global and local feature fusion sequence-aware ViT (GLF-ViT), modifying feature embedding to retain sampling point correlations and preserve more local information. By fusing global features from the classification token with local features from the encoder, the algorithm significantly enhances complex fault diagnosis. Experimental analyses on data segment length, network depth, feature fusion and attention head receptive field validate the approach, demonstrating that a shallower encoder network is better suited for high-dimensional time-series fault diagnosis in complex industrial processes compared to deeper networks. The proposed method outperforms state-of-the-art algorithms on the Tennessee Eastman (TE) dataset and demonstrates excellent performance when further validated on a power transmission fault dataset.

## 1. Introduction

In industrial process control, fault detection (FD) is crucial for ensuring system safety and reliability, making it an essential aspect of industrial production [1]. In recent years, data-driven fault diagnosis methods have attracted widespread attention due to their independence from complex physical models and strong system performance. These end-to-end methods achieve fault detection and diagnosis by analyzing the operational data of industrial systems, capturing the complex process variation features reflected in the data [2]. However, traditional FD methods face numerous challenges when dealing with the high-dimensional, nonlinear, and dynamic data present in industrial processes.

Statistical analysis is one of the earliest techniques used for fault diagnosis. Compared to model-driven fault diagnosis algorithms [3], methods such as principal component analysis (PCA) and independent component analysis (ICA) [4] can extract statistical features and analyze trends in the data, enabling anomaly detection to a certain extent. The non-dominated sorting genetic algorithm (NSGAII) [5] can be used for feature selection to identify the optimal subset of features for classification. Performance can be further enhanced by combining kernel PCA (KPCA) with kernel density estimation [6]. However, these methods typically assume that the data follow a certain distribution and require high-quality data preprocessing. For complex, multivariate chemical processes, the diagnostic accuracy and robustness of these methods are relatively limited.

With the advancement of machine learning techniques, increasing research has focused on applying machine learning algorithms to fault diagnosis. Traditional machine learning methods, such as support vector machines (SVM) [7] and random forests (RF) [8], achieve fault classification and diagnosis by learning various features in the data, particularly nonlinear features. These methods have demonstrated significant performance improvements; however, due to limitations in their structural design, they are unable to fully capture higher-order features and spatiotemporal dependencies, and their performance heavily relies on feature engineering and parameter optimization.

The rise of deep learning technology has provided new solutions for fault diagnosis. With the remarkable progress of convolutional neural networks (CNN) in computer vision, more researchers have applied deep learning algorithms from the image processing field to fault diagnosis. The application of image classification algorithms for time-series fault diagnosis can be broadly categorized into two approaches. One approach involves using feature extraction techniques to visually represent time-series data. For instance, Barrera-Llanga et al. used a visual geometry group (VGG) 19-based deep learning approach to transform current signals into spectral images for induction motor fault classification [9]. Similarly, Zhang et al. proposed a method combining frequency domain markov transition field (FDMTF) and a multi-branch residual convolutional neural network (MBRCNN) [10]. Another approach organizes multidimensional time-series data into graph structures for further processing. Since industrial process data have temporal and dimensional characteristics, they can be organized into a structure similar to images through slicing. By leveraging the powerful feature extraction capabilities of CNN, various local features within data segments can be automatically obtained [11,12,13]. To further extract multi-level high-level features, researchers have combined CNN with various algorithm structures, such as attention mechanisms [14], wavelet transforms [15], and auto-encoders [16], to further optimize feature weighting, combine time-frequency domain feature information, and train the network in an unsupervised manner to enhance classification performance and applicability. However, due to the limited receptive field of convolutional kernels, CNN-based algorithms lack the ability to capture temporal features. To address this, researchers have employed algorithms like 1D-CNN [17], dilated convolutional neural networks (DCNN) [18], temporal convolutional network (TCN) [19] to learn temporal variation features, and they have combined CNN with structures such as long short-term memory (LSTM) [20], gate recurrent unit (GRU) [21] and a deep shapley additive explanation (SHAP) [22,23] to effectively handle high-dimensional and complex time-series data, significantly improving the accuracy and robustness of fault diagnosis. However, these algorithms still rely on aggregating local features [24], and their ability to capture global temporal features in long-period, large-scale datasets needs further enhancement.

The Transformer model [25] has demonstrated exceptional performance in various fields, including natural language processing and time-series forecasting. Building on this, the vision transformer(ViT) [26] successfully adapted the Transformer architecture for image processing tasks, showcasing strong capabilities in capturing temporal features. This has made ViT a promising approach for fault diagnosis applications. Recent advancements have explored innovative modifications to the Transformer framework. For example, studies have CNN with Transformers to integrate the local feature extraction strengths of CNN with the global information modeling capabilities of Transformers [27,28]. Pyramid attention mechanisms have also been introduced, employing hierarchical structures to capture temporal dependencies across different scales [29]. Additionally, convolutional pooling and distillation operations have been applied between self-attention modules to downsample features and enhance feature representation [30], while pyramid encoder–decoder structures have been used to model multi-scale dependencies [31]. ViT retains most elements of the Transformer architecture while incorporating a classification token specifically designed for global feature learning and classification. Its potential has been explored in various applications, such as geological fault detection [32] and the diagnosis of rolling bearing faults in aircraft engines using model distillation techniques with multiple ViT models [33]. In the context of the TE process dataset, ViT has been evaluated alongside alternative approaches, including wavelet transforms and CNN, with its advantages in fault diagnosis being systematically analyzed [34].

Although the Transformer architecture has shown remarkable capabilities in sequence modeling, it faces several challenges when directly applied to high-dimensional time-series fault diagnosis. First, the standard Transformer lacks an explicit mechanism for global feature aggregation, which is crucial for fault pattern recognition across multiple sensor channels. Second, its purely sequential processing nature may not effectively capture the concurrent relationships among different sensor measurements. The ViT architecture, with its patch-based processing and classification token design, provides a more suitable framework for our scenario. The classification token serves as a natural aggregator for global feature learning, while the patch-based approach allows for more efficient parallel processing of multi-sensor data segments.

However, ViT was originally designed for image processing, and the high-dimensional time-series data in our study differs significantly from real image pixel values. The data points in these segments are often normalized values from sensors and lack the strong spatiotemporal relationships that traditional image pixels have with surrounding pixels. Simply applying image-based algorithms to high-dimensional time-series fault diagnosis may lead to information confusion, which can negatively impact system performance [34]. Additionally, increasing the depth of algorithms used for fault diagnosis does not necessarily improve recognition performance, which is quite different from image-based algorithms. When using ViT for image processing, global feature aggregation of image patches is necessary to effectively restore overall image semantics. However, when dealing with high-dimensional data, it is essential to specifically analyze the characteristics of the data being processed. This often requires recombining local features from each token and selecting appropriate network parameters to achieve maximum effectiveness.

Building on the limitations of previous studies, this research addresses the challenges of applying ViT to high-dimensional time-series data, particularly in fault diagnosis for the Tennessee Eastman chemical process. The main contributions of this paper are as follows:

- We propose a sequence-aware ViT network that is specifically adapted to high-dimensional sensor data, addressing the limitations of traditional ViT models when applied to data without inherent spatiotemporal relationships. This adaptation is critical for achieving accurate fault diagnosis in complex industrial processes.
- We enhance the fusion of global and local features by employing a multi-head attention mechanism. This approach improves diagnostic accuracy while maintaining a streamlined model design, avoiding additional structural complexity.
- We provide an in-depth analysis of attention focus across encoder layers, identifying potential causes for performance degradation in deeper networks. This analysis offers valuable guidance for designing more effective models in fault diagnosis, particularly for applications requiring high reliability.

The organization of this paper is as follows: Section 2 describes the relevant theoretical information for the algorithm. Section 3 introduces the embedding method and the global–local feature fusion approach proposed in this paper. Section 4 presents the TE dataset, compares the performance of the proposed algorithm in fault diagnosis with other state of the art (SOTA) algorithms, and analyzes the effects of parameters such as data length, encoder depth, and the number of attention heads on the algorithm’s performance. Section 5 concludes the paper with a summary and conclusion.

## 2. Preliminaries

### 2.1. Multi-Head Attention Mechanism

The multi-head attention mechanism is an extension of the self-attention mechanism. It enhances the model’s representation capacity, allowing the model to learn diverse features from different subspaces. Specifically, the multi-head attention mechanism computes attention through multiple distinct heads, concatenates the outputs of these heads, and then applies a linear transformation to generate the final output. The calculation process can be represented as:

(1) $$\mathrm{MSA}\left(Q,K,V\right)=\mathrm{Concat}\left({\mathrm{head}}_{1},{\mathrm{head}}_{2},\ldots ,{\mathrm{head}}_{h}\right)W^{O}$$

For each attention head, the calculation is as follows:

(2) $${\mathrm{Head}}_{i}=\mathrm{Attention}\left(QW_{i}^{Q},KW_{i}^{K},VW_{i}^{V}\right)$$

where $W_{i}^{Q},W_{i}^{K},W_{i}^{V}\in R^{d\times d_{k}}$, and $W^{O}\in R^{hd_{k}\times d}$ are learnable parameter matrices. The variable *h* represents the number of attention heads, and $d_{k}$ is the dimensionality of each attention head. The advantage of this structure lies in its ability to capture different feature representations in parallel through multiple attention heads, thereby enhancing the model’s capability to identify complex patterns. Additionally, the concatenation of attention results from multiple heads ensures that the final representation contains rich contextual information, which significantly helps improve the model’s generalization ability. As a result, the multi-head attention mechanism has become a core component of Transformer and its variants, widely applied in fields such as natural language processing and computer vision.

### 2.2. Positional Encoding

The primary purpose of positional encoding is to retain the sequential characteristics of the input data. It addresses the issue of preserving the inherent order information within the data after modeling. Positional encoding works by incorporating positional information into the input vectors, allowing the model to distinguish features at different positions within the sequence. A commonly used method for positional encoding is fixed positional encoding based on sine and cosine functions, with the formula as follows:

(3) $${\mathrm{PE}}_{\left(pos,2i\right)}=\sin\left(\frac{pos}{10000^{\frac{2i}{d}}}\right)$$

(4) $${\mathrm{PE}}_{\left(pos,2i+1\right)}=\cos\left(\frac{pos}{10000^{\frac{2i}{d}}}\right)$$

where ${PE}_{\left(pos,2i\right)}$ and ${PE}_{\left(pos,2i+1\right)}$ represent the encoding at position pos for the 2i-th and 2i+1-th dimensions, respectively, and *d* is the dimensionality of the encoding. This encoding method ensures that the relative positional information between different positions can be perceived by the model, enabling it to better handle the sequential relationships within the data. In the implementation of Transformer, positional encoding is directly added to the input embeddings, ensuring that each input vector contains positional information:

(5) $$X_{\mathrm{input}}=X_{\mathrm{embed}}+PE$$

where $X_{embed}$ represents the input vectors processed through the embedding layer, and PE is the positional encoding matrix. This combination allows the Transformer to leverage both the content information and the positional information when handling sequences, significantly improving the model’s performance in tasks that rely on sequence order.

### 2.3. Vision Transformer

ViT adapts the Transformer architecture for image classification by treating image patches as sequential inputs. Unlike traditional CNN-based approaches, ViT leverages the self-attention mechanism to capture global dependencies in images. Given an input image $x\in R^{H\times W\times C}$, ViT first divides it into *N* fixed-size patches (P×P), which are flattened into vectors. These vectors are then projected into a *D*-dimensional embedding space through a trainable linear projection $E\in R^{(P^{2}\cdot C)\times D}$:

(6) $$z_{p}^{i}=\mathrm{Flatten}\left(x\left[i\right]\right)E\in R^{D},\;i=1,2,\ldots ,N$$

To facilitate global feature learning, a learnable classification token $z_{\mathrm{cls}}^{0}\in R^{D}$ is prepended to the sequence of embedded patches. Additionally, positional encodings $E_{\mathrm{pos}}^{i}$ are added to each patch embedding to retain spatial information. The complete input sequence is formed as:

(7) $$Z_{0}=\left[z_{\mathrm{cls}}^{0};z_{p}^{1}+E_{\mathrm{pos}}^{1};\ldots ;z_{p}^{N}+E_{\mathrm{pos}}^{N}\right]\in R^{(N+1)\times D}$$

This sequence is processed through multiple Transformer encoder layers, each consisting of multi-head self-attention mechanisms and feed-forward networks. The encoder layers employ residual connections and layer normalization to ensure stable gradient propagation during training. In each layer, the self-attention mechanism enables each token to attend to all other tokens, capturing both local and global dependencies in the input. The final classification is performed by applying an MLP and softmax function to the classification token output from the last encoder layer:

(8) $$y=\mathrm{softmax}(\mathrm{MLP}\left(z_{\mathrm{cls}}^{L}\right))$$

where $z_{\mathrm{cls}}^{L}$ represents the final state of the classification token after *L* encoder layers. This architecture has demonstrated remarkable performance in various vision tasks, primarily due to its ability to model long-range dependencies and capture global context information.

## 3. Proposed Algorithm

In industrial fault diagnosis, unlike the traditional images that ViT mainly processes, the data in this study, although presented in image form, essentially consist of time-series data derived from various sensor readings. The pixels in these data lack the strong spatial relationships found in traditional images, but there is significant homogeneity between rows of pixels. Directly partitioning these data into patches, as in image processing, could disrupt this dependency. Therefore, we designed a new multidimensional time-series embedding method. In the Tennessee Eastman (TE) chemical production process, different faults are primarily associated with different state variables, and the time dependencies of each fault also vary. As a result, special attention is required when extracting features to ensure both depth and breadth. Overly deep feature extraction may lead to overfitting, which can degrade system performance.

**Figure 1:**
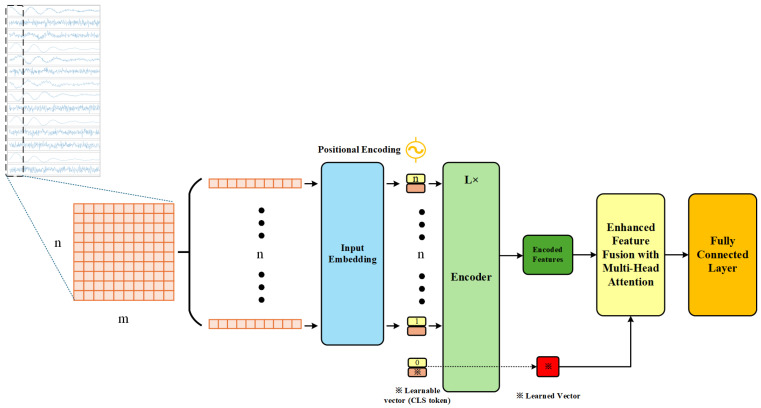
The structure of global and local feature fusion sequence-aware vision transformer (GLF-ViT). Industrial condition data collected by sensors is preprocessed and segmented into m×n matrices, then linearly projected by sampling points before being fed into the encoder for feature extraction and fusion, enabling fault classification.

The traditional ViT obtains more global information by stacking encoder layers and using larger datasets. However, for fault diagnosis in complex industrial processes, fault causes are often associated with multiple factors, and there are strong nonlinear, spatiotemporal correlations and couplings between faults. The combination of global and local information is crucial for achieving optimal fault diagnosis performance. Based on this, we apply a sliding step size of 1 for slicing the data to increase the data volume while reducing the number of stacked ViT encoder layers. Additionally, we introduce a multi-head attention mechanism to fuse the features learned by the classification token with the features output by the encoder. Combining the above improvements, we propose a global–local feature fusion ViT algorithm, with its structural flow shown in Figure 1. This approach fully leverages both global and local feature information, enhancing the algorithm’s fault diagnosis performance in complex industrial systems.

### 3.1. ViT Embedding for Multidimensional Time-Series

In the proposed algorithm, each sampling point of the multidimensional time-series segments is encoded. We model the temporal information and inter-feature relationships through high-dimensional feature mapping and the Transformer encoder. Let the input data segment be $X\in R^{n\times m}$, where *n* represents the sequence length (i.e., containing *n* sampling points) and *m* represents the number of features.

In the original ViT, the input image is first divided into fixed-size image patches, and each patch is then flattened and mapped into a high-dimensional space. This makes sense for images, as each patch maintains rich feature relationships. However, for our fault diagnosis scenario, we only use the data structure of images to process our multidimensional time-series data with multiple classifications. Directly segmenting the data would sever some relationships between variables, leading to information asymmetry and feature redundancy. Thus, in our method, there is no need to split the data segment into patches. Instead, the sequence of sampling points in each data segment is linearly projected to map the multidimensional features of all time steps into a unified high-dimensional space.

Specifically, we first project the input *m*-dimensional features into a *d*-dimensional feature space through a linear mapping $W\in R^{m\times d}$:

(9) Z=XW+b

where $Z\in R^{n\times d}$ represents the high-dimensional feature representation after projection, and $b\in R^{d}$ is the bias term. Unlike the patch-based division in ViT, our input retains the original time-series length *n*, enabling us to directly capture the global temporal information of the sequence. We then add a learnable classification token and positional encoding before feeding the data into the encoder to begin feature extraction.

### 3.2. Local Feature Extraction

For the projected time-series representation $Z\in R^{(n+1)\times d}$, the local temporal features are extracted through a carefully designed attention mechanism in the encoder layers. Given $z_{t}\in R^{d}$ representing the projected features at time step *t*, the self-attention mechanism processes each time step while considering its relationships with other time points in the sequence.

The attention mechanism in each encoder layer computes the local temporal features through:

(10) $$h_{t}^{l}=\sum_{i=1}^{n}\alpha_{ti}^{l}z_{i}^{l-1}$$

where $h_{t}^{l}$ represents the features of time step *t* at layer *l*, and the attention weight $\alpha_{ti}^{l}$ is computed as:

(11) $$\alpha_{ti}^{l}=\mathrm{softmax}\left(\frac{{\left(W_{Q}^{l}z_{t}^{l-1}\right)}^{T}\left(W_{K}^{l}z_{i}^{l-1}\right)}{\sqrt{d_{k}}}\right)$$

with $W_{Q}^{l},W_{K}^{l}\in R^{d\times d_{k}}$ being the query and key projection matrices at layer *l*.

This computation effectively models what we term “cross-temporal features”—the dynamic relationships between different time points in the sequence. As each time step’s features are computed by attending to all other time steps, the model captures both local temporal patterns and their evolution throughout the sequence. The continuous changes in multidimensional relationships over time are inherently modeled, allowing the capture of cross-dimensional features based on temporal dependencies.

While deeper networks are often preferred in image-based ViT applications to compensate for information loss from patch division, industrial process data present different challenges. The temporal variability, strong coupling between variables, and nonlinear characteristics of industrial processes require a more nuanced approach. Different fault patterns often manifest in varying clusters of variables and across different temporal scales. Therefore, we intentionally design our architecture with fewer encoder layers (L≤4) but leverage multi-head attention mechanisms with differentiated receptive fields:

(12) $$h_{t}^{l}=\mathrm{Concat}\left({\mathrm{head}}_{1}^{l},...,{\mathrm{head}}_{h}^{l}\right)W_{O}^{l}$$

where each attention head can specialize in capturing different aspects of the temporal patterns. This approach enriches the feature representation while maintaining the temporal integrity of the data, better suiting the characteristics of industrial fault diagnosis.

The high-dimensional features output by the encoder $H=[h_{1},...,h_{n}]$ (shown as dark green parts in Figure 1) predominantly contain local temporal features, which will be further enhanced through global–local feature fusion in the subsequent stage.

### 3.3. Global–Local Feature Fusion

The key difference between ViT and the traditional Transformer is the introduction of a learnable classification matrix, the classification token. While the Transformer also captures global information for each token, it primarily focuses on the output features of individual tokens. In contrast, the classification token uses the attention mechanism to gather features from all tokens for classification. For most fault detection tasks, the features obtained by the classification token are sufficient. However, some complex industrial faults have intricate causes that require distinguishing across multiple variables with cross-dimensional and cross-temporal features. To address this issue, we employ a multi-head attention mechanism (the light yellow parts in Figure 1) to further fuse the global features obtained by the classification token (the red parts in Figure 1) with the local features output by the encoder. This deep fusion maximizes the utilization of both types of features, enhancing the system’s ability to handle complex industrial faults by leveraging the combination of diverse features. Assuming the input sequence after linear mapping and positional encoding is $Z\in R^{(n+1)\times d}$, where *n* represents the number of segments into which the input data are divided, and *d* represents the feature dimensionality of each segment. The sequence includes one classification token $z_{\mathrm{CLS}}$ and *n* feature segments $z_{i}$, i.e.,

(13) $$Z=\left[z_{\mathrm{CLS}},z_{1},z_{2},\ldots ,z_{n}\right]\in R^{(n+1)\times d}$$

This sequence is fed into the Transformer encoder for feature extraction:

(14) $$H=T\left(Z\right)\in R^{(n+1)\times d}$$

The output *H* of the encoder contains the classification token output $h_{\mathrm{CLS}}$ and the high-dimensional representations $h_{i}$ of all the feature segments after processing through multiple layers of self-attention and feed-forward networks:

(15) $$H=[h_{\mathrm{CLS}},h_{1},h_{2},\ldots ,h_{n}]$$

where $h_{\mathrm{CLS}}\in R^{d}$ is the classification token output from the encoder, representing global features, and $h_{i}\in R^{d}$ represents the encoded features of the *i*-th time segment. The classification token output $h_{\mathrm{CLS}}$ interacts with the encoder feature maps $h_{i}$ through the multi-head attention mechanism, resulting in fused features that contain both global and local information.

Assuming the query (*Q*) in the multi-head attention mechanism comes from the classification token, while the key (*K*) and value (*V*) come from the encoded feature maps:

(16) $$\begin{array}{cc} Q & =h_{\mathrm{CLS}}\in R^{1\times d} \end{array}$$

(17) $$\begin{array}{cc} K & =V=\left[h_{\mathrm{CLS}},h_{1},h_{2},\ldots ,h_{n}\right]\in R^{n\times d} \end{array}$$

The multi-head attention mechanism maps the query, key, and value to a lower-dimensional space through linear projections and computes the attention weights:

(18) $$Q=W_{Q}h_{\mathrm{CLS}},\;K=W_{K}G,\;V=W_{V}H$$

where $W_{Q}$, $W_{K}$, $W_{V}\in R^{d\times d_{k}}$ are the trainable linear projection matrices, and $d_{k}$ is the dimensionality of the lower-dimensional space. The attention weights are computed using the Softmax function:

(19) $$\mathrm{Attention}\left(Q,K,V\right)=\mathrm{softmax}\left(\frac{QK^{T}}{\sqrt{d_{k}}}\right)V$$

$h_{\mathrm{fusion}}$ is the representation that combines global and local features, and it is passed through a fully connected layer and a softmax layer for classification:

(20) $$y=\mathrm{softmax}(W\cdot h_{\mathrm{fusion}}+b)$$

where $W\in R^{C\times d}$ is the weight matrix of the classification layer, $b\in R^{C}$ is the bias term, and *C* represents the number of classes.

The fusion process through multi-head attention mechanism provides several key advantages in our approach. First, by using $h_{\mathrm{CLS}}$ as the query and encoder features as both key and value matrices, we enable the model to learn selective feature aggregation based on the global context. Each attention head can specialize in different aspects of the temporal patterns: some heads focus on short-term dynamics by attending to temporally adjacent features, while others capture long-range dependencies by attending to features across the entire sequence. This multi-scale feature learning is particularly crucial for industrial fault diagnosis, where fault patterns may manifest at various temporal scales. Second, the attention weights learned during the fusion process effectively serve as an adaptive feature selection mechanism, allowing the model to emphasize the most relevant temporal patterns for different fault types. Finally, this fusion architecture maintains the integrity of both global and local features throughout the process, as the original features from both the classification token and encoder output participate in the attention computation without information loss. This comprehensive feature utilization significantly enhances the model’s capability to handle complex industrial faults that exhibit both global trends and local anomalies.

## 4. Case Study

This study employs both the Tennessee Eastman dataset and a power system transmission fault dataset for case studies. The TE dataset, with its numerous variables and diverse fault types, enables comprehensive analysis and parameter exploration. Meanwhile, the power system transmission fault data serve to validate the algorithm’s applicability, further demonstrating its effectiveness.

### 4.1. TE Database

**Figure 2:**
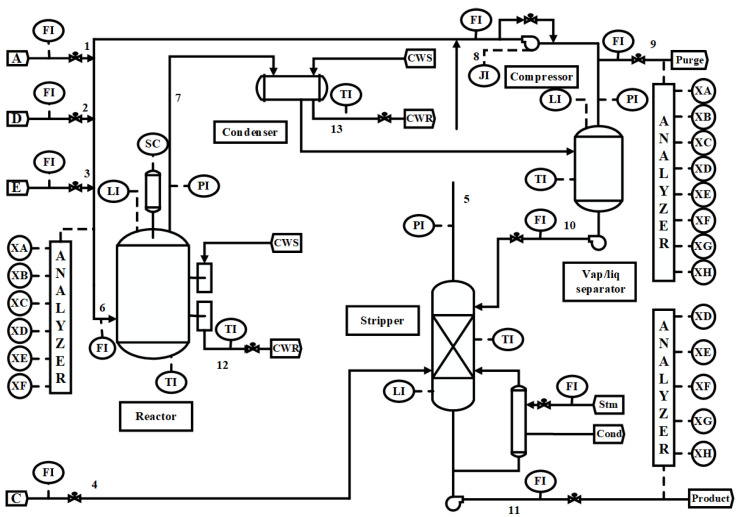
Tennessee Eastman test problem [35].

The TE database is a classic benchmark dataset used in the research of industrial process fault detection and diagnosis. This dataset was first introduced by Downs and Vogel in 1993 to simulate the dynamic behavior and fault scenarios of real chemical processes (as shown in Figure 2), allowing for the evaluation of fault detection methods. The TE database’s simulation environment replicates a chemical reaction process from Eastman Chemical Company, involving multiple operating units and control loops, reflecting the complexity and uncertainty found in real industrial environments.

In this paper, we use the TE dataset [36] to test and validate the performance of the proposed algorithm. The dataset contains 41 measurable process variables and 11 control variables, covering various aspects such as temperature, pressure, flow rate, and concentration. Each variable has its own operating range and dynamic characteristics. The simulation data include normal operation data as well as 20 different types of fault data. These fault types encompass various scenarios, including equipment faults, process control faults, and external disturbances.

The TE database, provided by Harvard University, is divided into training and testing sets, both of which include fault and normal data. For ease of data management, we used the normal and fault data from the training set in our experiments. The training set fault data simulate 500 runs, each simulating 20 fault conditions. Each fault condition runs for 25 h, with the fault introduced after one hour of normal operation, and data sampled every three minutes. Each fault type has 500 data points per run. The training set normal data also consist of 500 runs, each running for 25 h with the same sampling frequency as the fault data, providing 500 normal data points per run.

### 4.2. Data Preparation and Preprocessing

Since the fault is introduced after the first hour in each run of the fault data, we preprocessed the data by selecting the 21st to 500th data points for our experiments. To ensure consistency in the time process and facilitate data handling, we also removed the first 20 data points from each run of the normal data. To fully utilize the potential of the algorithm, we used all 500 simulation runs in the experiment. To maintain the integrity of the temporal characteristics, we randomly selected runs for splitting the dataset into training, validation, and testing sets. Specifically, 460 runs were used for training, 20 runs for validation, and 20 runs for testing. As a result, the training set contains a total of 4,636,800 data points, while the validation and test sets each contain 201,600 data points.

**Figure 3:**
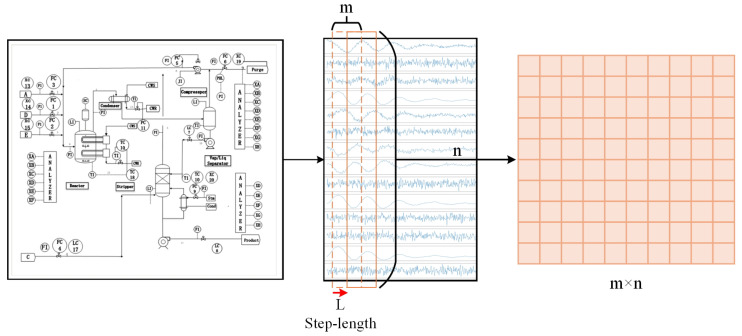
Data slicing process. Slicing the *n*-dimensional data using a length of *m* sampling points with a step size of *L*.

We then segmented the time-series data from the TE database as Figure 3. Before segmentation, the data were normalized according to the type of variable. Each data segment consists of *n* sets of data and includes *m* process variables (where m=52 in the TE database). This segmentation method allows us to capture the dynamic interactions between variables and the temporal dependencies, providing strong support for feature extraction in the subsequent model stages. To ensure the correlation between data segments, we used a sliding window approach with a step size of l=1. This sliding window technique maintains data continuity while increasing the number of training samples, thereby improving the model’s generalization ability. For a given time-series $\left\{x_{t}|t=1,2,\ldots ,T\right\}$, where $x_{t}\in R^{m}$ represents the *m* variable values at time *t*, data segments are generated through the sliding window. Assuming each data segment has a length of *n*, the *i*-th data segment can be represented as:

(21) $$X_{i}=\left[x_{i},x_{i+1},\ldots ,x_{i+n-1}\right],\;i=1,2,\ldots ,T-n+1$$

where, $X_{i}\in R^{n\times m}$ represents a data segment containing *n* time steps and *m* variables. Through this method, the original time-series is transformed into a series of sequences, each of length *n*. These data segments serve as input to the deep learning model for fault detection and classification tasks. To investigate the impact of input sequence length on the model, we extracted segments of four different lengths: n=5,10,20,40 for subsequent experimental analysis. To ensure that the data within each segment remains temporally continuous, segmentation was performed on a per-run basis, without crossing runs. This approach ensures that all data within a segment comes from a single process and maintains temporal continuity, preserving the cross-temporal feature information as much as possible. Table 1 shows the number of data segments obtained for different segment lengths.

### 4.3. Model Training Parameters

Model optimization is achieved by minimizing the cross-entropy loss function:

(22) $$L\left(\Phi \right)=-\frac{1}{N}\sum_{i=1}^{N}\sum_{k=1}^{K}t_{ik}\log p\left(\hat{y_{i}}=k|X_{i};\Phi \right)$$

where *L* is the loss function, Φ is the set of trainable parameters, *N* is the number of training samples, $t_{ik}$ is an indicator function for sample *i*’s true label in class *k* (with a value of 0 or 1), and $p(\hat{y_{i}}=k|X_{i};\Phi )$ is the probability predicted by the model that $X_{i}$ belongs to class *k*.

The proposed algorithm utilizes a four-layer encoder with eight attention heads per encoder. The feature fusion mechanism employs 32 attention heads in the multi-head attention module, and the linear projection dimension is set to 128. The dropout rate is configured at 0.1. The model is trained using the Adam optimizer, with a learning rate set to 0.00001, and mixed precision training is employed to improve computational efficiency. Additionally, a learning rate scheduler with linear warmup and cosine annealing is used, where the first 5% of training steps are dedicated to warmup, allowing for a smoother adjustment of the learning rate. The batch size is set to 1024, and the model is trained for a total of 600 epochs. To prevent overfitting, an early stopping mechanism is implemented, terminating the training if the validation accuracy does not improve for 50 consecutive epochs. The experiments were conducted on the PyTorch platform, using a server equipped with four NVIDIA Tesla V100-SXM2-32GB GPUs located in Zhengzhou, China.

From the perspective of time complexity, the algorithm consists of three main components: the input projection layer, the multi-head self-attention mechanism, and feature fusion. For an input sequence of length *n* and feature dimension *d*, the complexity of the input projection is O(nd). In the *L*-layer Transformer encoder, the self-attention mechanism in each layer has a complexity of $O(n^{2}d)$, with the major computational cost arising from the matrix multiplication of query-key pairs. During the feature fusion stage, the interaction between the classification token and the feature map is implemented using a multi-head attention mechanism, which also has a complexity of $O(n^{2}d)$. Thus, the overall time complexity of the algorithm is $O(Ln^{2}d)$, where the primary computational bottleneck lies in the quadratic complexity of the self-attention mechanism. From the perspective of space complexity, the model parameters are primarily composed of the Transformer encoder parameters $O(Ld^{2})$ and the input projection layer parameters O(nd). During runtime, the storage requirements include the attention matrix $O(n^{2})$ and the intermediate feature representations O(nd). Considering a batch size of *b*, the actual memory cost during training and inference is $O(bn^{2}+bnd)$. Overall, the algorithm achieves efficient computation while maintaining high performance, particularly when the sequence length and feature dimensions are within a moderate range.

### 4.4. Performance Comparison with Other SOTA Algorithms

**Figure 4:**
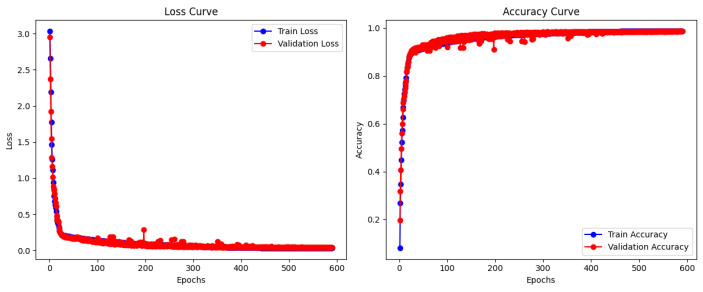
Training loss and validation accuracy curves.

To comprehensively evaluate the performance of our algorithm, we employed three widely used metrics: recall, precision, and F1 score. We compared our method with several state-of-the-art (SOTA) algorithms that have shown excellent performance in recent years, including the IPO-ViT [34] based on an improved ViT, the Target Transformer [37] specifically designed for fault diagnosis in chemical processes, the LGS-CNN [17] that cleverly combines local and global features under one-dimensional convolution, and the innovative CNN-LSTM that integrates CNN with LSTM. Figure 4 presents the training loss values and validation accuracy during the training process. The results in Table 2 demonstrate that our method outperforms all other algorithms across all metrics, particularly for challenging fault categories such as faults 3, 9, and 15, where significant improvements are observed. The recall for fault 3 and 15 substantially increased to 99.35% and 96.24%, while the precision for faults 9 improved to 97.15%, respectively. Moreover, the performance metrics for other faults all exceeded 98%, with 11 faults achieving 100% across all three key indicators, highlighting the exceptional performance of our algorithm. We attribute these advantages primarily to the following factors: the introduction of a self-attention mechanism to better capture long-range dependencies and global contextual information in time-series data; the innovative design of a global–local feature fusion to simultaneously utilize data representations at different scales and granularities; and the targeted improvement and optimization of the ViT structure, such as adopting per-time-step embedding and multi-head attention to better adapt to the characteristics of time-series data. The extensive experimental results and detailed comparative analysis demonstrate that our algorithm achieves significant performance improvements on the TE dataset, showcasing its immense application potential in the field of intelligent fault diagnosis for complex industrial processes.

**Figure 5:**
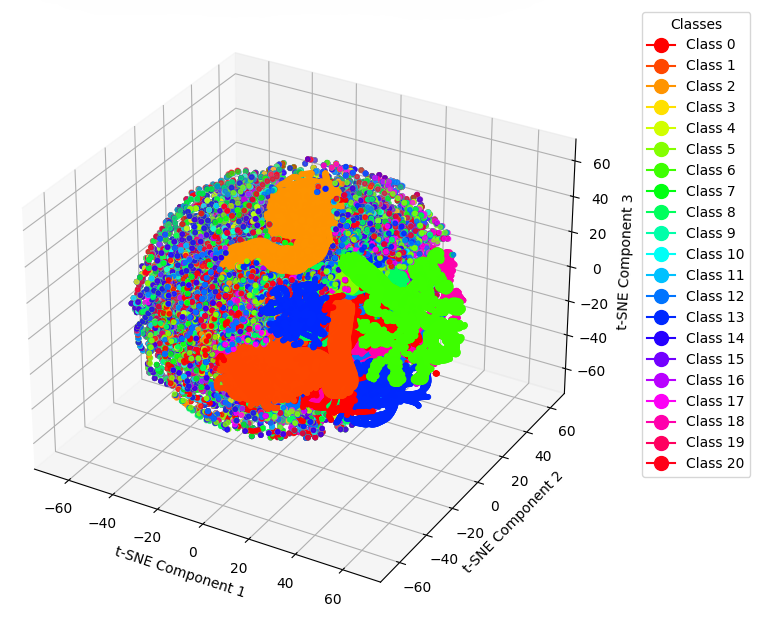
t-SNE visualization of test set data before inputting into GLF-ViT.

**Figure 6:**
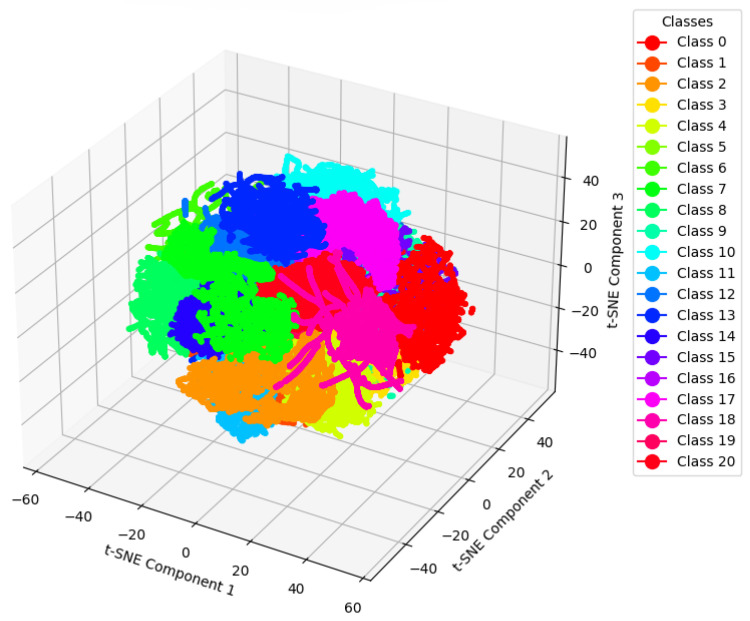
t-SNE visualization of test set data before the classification layer in GLF-ViT.

We used t-SNE for dimensionality reduction to visualize the raw data. After applying dimensionality reduction to the test set data as shown in Figure 5, it is evident that the raw data are highly entangled, with multiple categories intertwined. Faults 3, 9, and 15 are almost completely mixed together, and the raw data are characterized by a large number of categories and complex distributions. However, after applying our proposed algorithm in Figure 6, the chaotic and complex data have been organized into several distinct states, with each color block representing a specific state. The analysis of t-SNE visualization reveals the mechanism by which the GLF-ViT model captures key features and contributes to improved diagnostic performance. The raw data, prior to model processing, exhibit a highly entangled distribution, particularly for faults 3, 9, and 15, which overlap significantly with other categories, making effective classification challenging. After processing with the GLF-ViT model, the feature distribution becomes well structured, with distinct clusters for each category, and the features of complex faults 3, 9, and 15 appear more cohesive and concentrated. This demonstrates that the model effectively enhances the extraction of cross-dimensional and temporal features through the classification token for global feature learning and the encoder’s mechanism for preserving local features.

The proposed GLF-ViT demonstrates significant advantages over existing state-of-the-art methods, such as IPO-ViT and CNN-LSTM. Specifically, it optimizes the embedding process for high-dimensional time-series data, avoiding information loss and redundancy caused by direct data segmentation. Through an innovative global–local feature fusion strategy combined with multi-head attention mechanisms, the algorithm effectively captures cross-dimensional and temporal dependency features. Additionally, it employs a shallow encoder network to mitigate the performance degradation often associated with deeper networks, making it more suitable for feature extraction in complex industrial processes.

### 4.5. Impact of Segment Length on Performance

The selection of data segment length is critical in multidimensional time-series processing, as it affects the temporal features the algorithm can capture. Our analysis using various segment lengths (5, 10, 20, 40) demonstrated that longer data segments generally improved recognition performance. However, excessively long segments could decrease the effectiveness of certain faults due to the inclusion of redundant features, impacting the algorithm’s robustness.

We adopted a three-layer encoder stacking structure, combining feature fusion with the multi-head attention mechanism, where the number of attention heads was set to 16. As shown in Table 3, the faults 8, 10, 11, 12, 13, 16, 17, 18, and 20 benefited from longer segment lengths, showing improved recall and precision due to their strong temporal dependencies. For easier-to-recognize states such as the normal condition and faults 1, 2, 4, 5, 6, 7, 14, and 19, high recognition rates were maintained across all segment lengths, indicating low temporal dependencies. However, some faults like 12 displayed diminishing returns in recognition as the segment length increased beyond a certain point, indicating that overly long segments may introduce irrelevant information. Faults 18 and 20 showed fluctuations in recognition performance across different segment lengths, highlighting their complexity and the variable effectiveness of extracted features.

**Figure 7:**
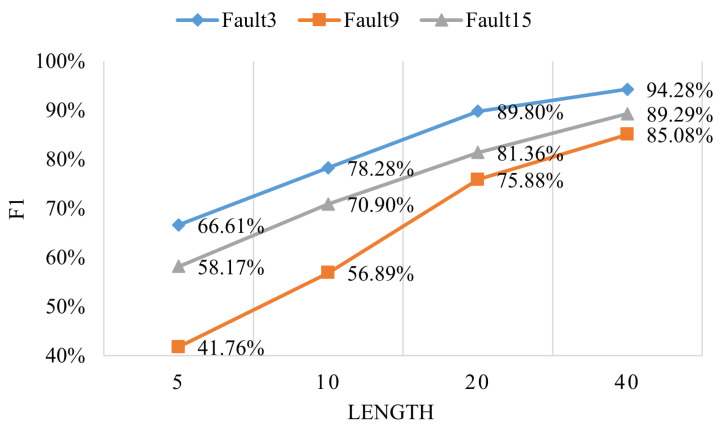
The correlation between the F1 score and the data segment length for faults 3, 9, and 15.

For the challenging faults 3, 9, and 15, the proposed global–local feature fusion mechanism effectively enhances classification performance by precisely modeling the unique characteristics of these faults. Analysis of data segment length reveals that the complexity of faults 3, 9, and 15 arises from their highly coupled temporal dependencies and nonlinear inter-variable features. Shorter time segments fail to provide sufficient contextual information to capture these features, whereas longer segments effectively capture cross-temporal feature correlations. For example, when the segment length increases from 5 to 40, the F1 score for fault 3 improves significantly from 66.61% to 94.28% as shown in Figure 7, with similar performance gains observed for faults 9 and 15. This demonstrates that increasing the segment length enables the model to extract key features from complex temporal patterns more comprehensively, thereby improving its diagnostic capability for these faults.

### 4.6. Analysis of Internal Structural Variations

To investigate the impact of the number of encoder layers and the number of attention heads in the feature fusion mechanism on performance, we designed different structures for validation experiments. Based on the previous analysis of data segment lengths, we selected the best-performing segment length of 40 as the input length. From Table 4, it is evident that when the number of encoder layers is 4 and the number of attention heads in the multi-head attention mechanism is 32, the system achieves the best performance. When the algorithm is configured with a three-layer encoder, the total parameter count amounts to 1,854,741. For a four-layer encoder, the parameter count increases to 2,447,765, and for a five-layer encoder, it reaches 3,040,789.

We discovered an interesting phenomenon: system performance did not improve with an increase in the number of encoder layers or attention heads in the multi-head attention mechanism. On the contrary, blindly increasing network depth led to a decline in performance. The high-dimensional temporal characteristics of industrial process data pose unique challenges for feature extraction. Unlike traditional image data, industrial data exhibit both short- and long-term dependencies, along with significant local sparsity and nonlinear coupling between variables. Shallow networks can effectively balance the extraction of global information and critical local features through a dispersed attention range. However, as the encoder depth increases, the attention mechanism tends to focus on longer time spans, overly emphasizing global information while neglecting local dependencies, which leads to an imbalance in feature representation. This issue is particularly pronounced for complex faults such as faults 3, 9, and 15, which rely on short-term temporal features; deeper networks fail to adequately capture these critical characteristics, thereby negatively affecting diagnostic performance. Additionally, deeper networks are more prone to feature redundancy and overfitting. While shallow networks focus on extracting sufficient global and local features, deeper networks often introduce redundant features that fail to provide additional useful information and may amplify noise in the data, thereby weakening generalization capability. These findings highlight the need to strike a balance between network depth and redundancy in designing models for industrial data, as shallow networks not only avoid feature redundancy but also better adapt to the diverse temporal dependencies of such data, resulting in superior diagnostic performance.

To further investigate the impact of the number of attention heads in the encoder mechanism on algorithm performance, we conducted experiments using a four-layer encoder structure with different numbers of attention heads (8, 16, 32). The attention heads in the fusion mechanism were fixed at 32, and other settings remained the same. The results show that the F1 scores for 8, 16, and 32 attention heads were 98.37%, 98.33%, and 98.22%, respectively. It can be observed that increasing the number of attention heads did not improve performance and instead led to a slight decrease. Moreover, a higher number of attention heads significantly increases algorithmic complexity. Considering these factors, we ultimately set the encoder network’s attention head count to 8.

### 4.7. Ablation Study

**Figure 8:**
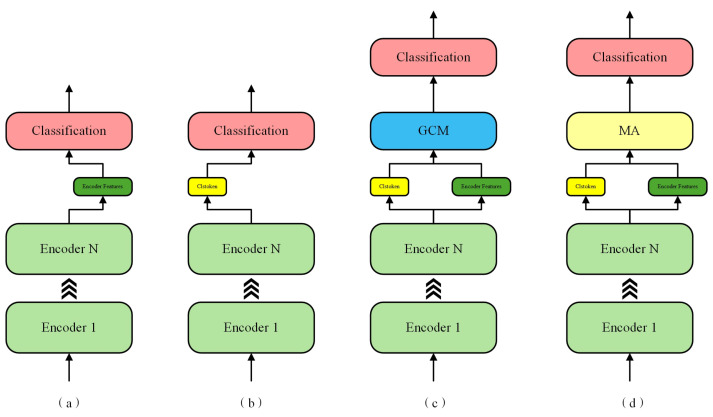
Four variant structure diagrams: (**a**) classification using only encoder features (Transformer), (**b**) classification using only classification token features (ViT), (**c**) feature fusion using a gating mechanism, (**d**) the proposed algorithm structure.

To more effectively analyze and verify the effectiveness of our algorithm, we designed several variants of the existing algorithms. Their structures are shown in Figure 8. This allows for a clearer understanding of the decision-making basis and the effects of feature fusion when dealing with complex industrial chemical process data. For the experiments, we used a four-layer encoder stack, with feature fusion performed using a 16-head multi-head attention mechanism. The F1 scores of the four algorithm variants are presented in Table 5.

The main difference between variants a and b lies in the features used for classification. Variant a uses the features output by the encoder, while variant b utilizes the features from the learnable classification vector. The learnable classification vector learns to interact with all segmented embedding blocks through the attention mechanism, thus incorporating global information. However, the output of the features by the encoder still retains a significant amount of information based on the embedding segments. Unlike traditional ViT, we are working with industrial data, where the data are grouped by segment length before entering the encoder and further split and mapped into a high-dimensional space based on sampling points. Each sampling point’s data contain critical feature information, and these key features play an essential role in detecting complex faults, such as faults 3, 9, and 15 in the TE dataset. From the results, we can see that structure b performs slightly better overall. However, in detecting certain faults, structure a shows some advantages. This validates our previous analysis: when applying ViT-based algorithms to multidimensional time-series industrial data, it is necessary to combine the advantages of both the learnable classification vector and the encoder output features to improve recognition performance.

In addition, we removed the positional encoding component from the algorithm for comparison. Experimental results show that, compared to the same algorithm with positional encoding, the version without positional encoding saw its recall, precision, and F1-score drop to 96.93%, 96.94%, and 96.81%, respectively, indicating a notable performance decline. This suggests that positional encoding provides valuable temporal information, enabling the algorithm to more effectively handle multivariate time-series fault diagnosis.

To analyze the feature fusion process, we compared the proposed algorithm’s multi-head attention mechanism with a gating mechanism. The gating mechanism functions like a switch, computing a set of weights to determine the fusion ratio between two inputs. The gated weights are computed through a fully connected layer using the classification vector and the full set of encoder output features. These weights are then compressed into the range of [0, 1] using the sigmoid activation function, and the two inputs are combined proportionally to produce fused features for classification. In contrast, multi-head attention calculates the correlations between input features and combines the outputs of multiple attention heads to complete complex feature fusion. Each attention head models the relationships between features in different subspaces, allowing it to capture both global and fine-grained dependencies more effectively. The comparison results show that the proposed algorithm performs better. This could be because the gating mechanism is a linear weighted method, making it difficult to capture complex relationships and high-order dependencies between input features. The multi-head attention mechanism, on the other hand, excels at capturing long-range dependencies when handling complex data, giving it greater flexibility and stronger expressive power in feature fusion, which leads to better performance.

The multi-head attention mechanism plays a critical role in the deep fusion of global and local features, which is essential for classifying faults 3, 9, and 15. These faults are characterized by highly complex variable coupling and nonlinear temporal relationships. The classification token captures global information through interactions with all time segments, while the encoder outputs retain local features at each time step, ensuring the integrity of fine-grained information. By leveraging multi-head feature fusion, the attention mechanism effectively extracts cross-dimensional and cross-temporal dependencies inherent in these faults. Notably, the shallow network structure and the dispersed coverage of attention heads provide highly adaptive feature extraction capabilities for faults 3, 9, and 15. In contrast, deeper networks may introduce feature redundancy, potentially undermining the model’s ability to identify these faults. Therefore, the proposed fusion mechanism precisely captures the essential characteristics of these complex faults, achieving accurate classification for faults 3, 9, and 15, and offering strong support for fault diagnosis in complex industrial processes.

### 4.8. Analysis of Attention Head Receptive Field

To better explore what the attention mechanism in our improved algorithm focuses on, we analyzed the attention scope of each layer’s attention mechanism in the encoder. Each attention head in the encoder generates a weight matrix. According to the algorithm’s configuration, the optimal data segment length is 40, resulting in a total of 41 tokens. The attention mechanism generates a 41 × 41 weight matrix. From this weight matrix, we can understand the degree of focus each time point has relative to the others. If there are many non-zero or high-weight values in the matrix, it indicates that the attention mechanism is focusing on many time points.

**Figure 9:**
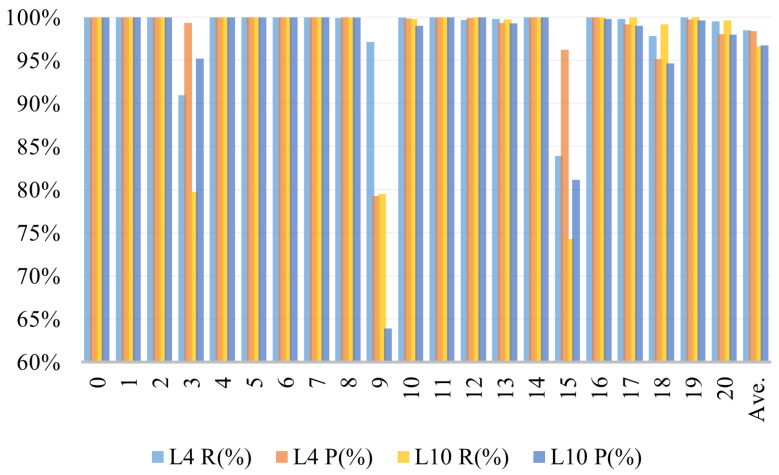
Precision and recall of the GLF-ViT algorithm with 4-layer and 10-layer encoders.

We normalized the weights of each attention head, ensuring the total weight sums to 1, and then set a threshold to determine whether a time point is being focused on. Here, we set the threshold at 0.02. We then averaged the number of time points that each attention head focused on, which gives us the attention scope for each head. We trained both the 4-layer encoder and the 10-layer encoder models for comparison, using data segments of length 40, with all other parameters kept the same. The recognition results of the two models are shown in Figure 9.

**Figure 10:**
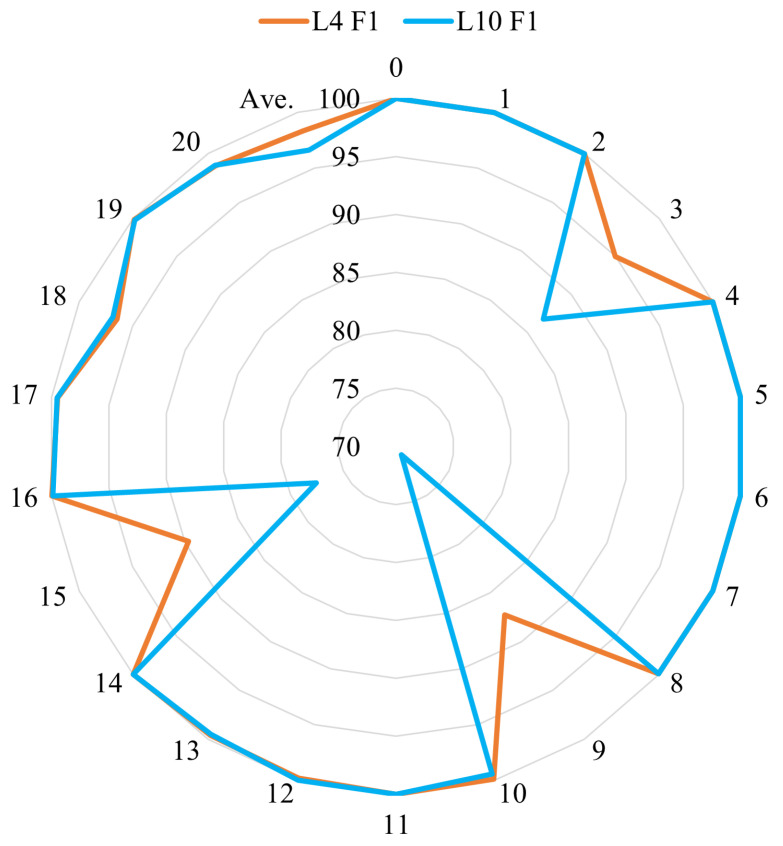
Radar chart of F1 scores for the GLF-ViT algorithm with 4-layer and 10-layer encoders.

In terms of recognition performance, the 4-layer model achieved an average precision of 98.50% and an average recall of 98.38%, both higher than the 10-layer model’s average precision of 96.75% and recall of 96.65%. This indicates that increasing the depth of the model does indeed lead to a decline in algorithm performance. When examining the performance for individual fault categories, the main differences are observed in faults 3, 9, and 15, which are known to be difficult to diagnose. The 4-layer model’s precision and recall for these faults were significantly higher than those of the 10-layer model. In other words, as the number of encoder layers increases, the system’s diagnostic performance for these three faults degrades. To provide a more intuitive analysis of the specific performance changes, we have plotted radar charts of the F1 scores for both algorithms for comparison in Figure 10.

The radar chart provides a clearer visualization of the overall performance of the two models across faults, excluding faults 3, 9, and 15. Both models performed well, with F1 scores above 95% for most states and even reaching 100% for several categories, indicating that the proposed algorithm offers strong diagnostic capability for the TE chemical process as a whole. Comparatively, the F1 scores of the 4-layer model were either equal to or better than those of the 10-layer model, especially for faults 3, 9, and 15, where the 10-layer network showed significant performance degradation. In the 10-layer network, the F1 score for fault 3 dropped to 86.76%, an 8.21% decrease compared to the 4-layer model. For fault 9, the F1 score was 70.84%, a decrease of 16.45%, and for fault 15, the F1 score was 77.55%, a drop of 12.12%. These reductions in performance for these three faults were the primary reason for the overall diagnostic performance decline in the 10-layer network. However, for faults 12 and 18, the 10-layer network showed slight improvements compared to the 4-layer network. The F1 score for fault 12 increased from 99.79% to 100%, a 0.21% improvement, and for fault 18, the F1 score rose from 96.45% to 96.84%, an increase of 0.39%. Although these performance gains were limited, they demonstrate that the causes of faults in chemical process multi-class problems are complex. The main factors driving each fault, along with their variable and temporal dependencies, vary, and adjustments to the algorithm can have differing effects on diagnostic performance across different states. We will further investigate these causes through an analysis of the attention mechanism.

**Figure 11:**
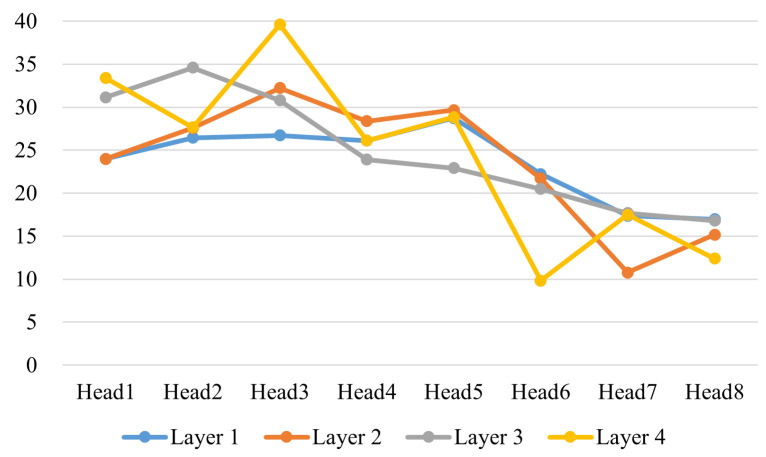
The attention head receptive field for the GLF-ViT algorithm with the 4-layer model.

**Figure 12:**
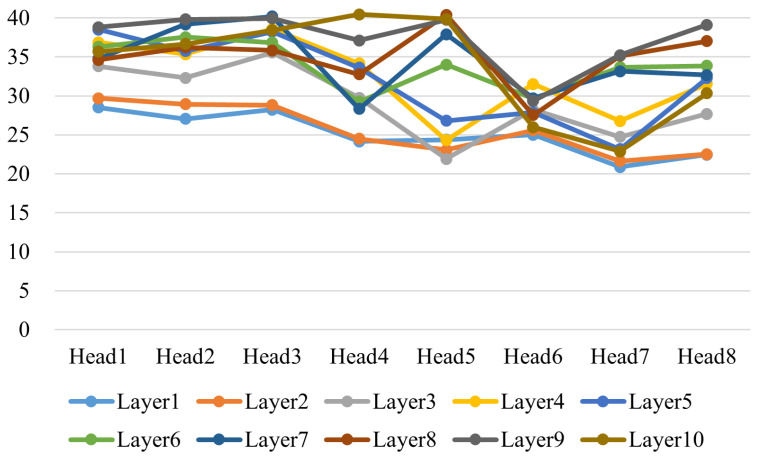
The attention head receptive field for the GLF-ViT algorithm with the 10-layer model.

From Figure 11, we can clearly observe that the attention heads in the 4-layer encoder cover a range of 10 to 40 sampling points, with varying lengths and significant differences in attention distribution. In contrast, the attention heads in the 10-layer encoder focus on ranges above 20 sampling points in Figure 12, mostly concentrated around 30 points. We speculate that this difference in the attention range contributes to the performance disparity. In the TE process, faults exhibit varying degrees of correlation not only with different sensors but also with temporal dependencies. The broader range of attention distributions in the 4-layer model appears to suit fault diagnosis in the TE process better. By combining this analysis with recognition performance, we can infer that a diverse range of attention spans allows the model to better distinguish faults 3, 9, and 15. These traditionally challenging faults do not seem to exhibit long temporal correlations. If all attention heads focus on ranges beyond 20 sampling points, it may interfere with diagnosing these faults.

Moreover, the attention span differences further explain why a data segment length of 40 achieves better performance—shorter data segments fail to provide sufficient temporal dependencies. Some faults, such as faults 12 and 18, may require longer temporal relationships, but longer attention spans alone may not be the main factor for fault identification, as extended attention does not significantly improve performance for these faults. Additionally, the analysis of the attention field diagram shows that shallow networks, with their dispersed attention distribution, are particularly effective in capturing short- and mid-term dependency features, while the classification token ensures comprehensive modeling of the overall fault patterns by extracting global features. This fusion mechanism of global and local features significantly improves the distinction between categories and enhances diagnostic accuracy, providing strong support for identifying complex faults.

The disparity in attention spans also clarifies why traditional Transformer and ViT models do not exhibit improved performance with deeper layers when applied to high-dimensional time-series-to-image fault diagnosis. Ultimately, this is because the images generated from high-dimensional time-series data differ fundamentally from the images used in computer vision tasks. Unlike traditional images, where there are strong spatial and temporal relationships between pixels, the transformed images of time-series data do not exhibit such properties. As a result, deeper networks may cause feature redundancy, leading to performance degradation. When migrating image classification algorithms to the fault diagnosis domain, it is essential to consider the nature of the data, the causes of faults, and the characteristics of the transformed images. This approach ensures the best performance and enhances the algorithm’s applicability in fault diagnosis.

### 4.9. Power System Transmission Line Fault Detection Verification Experiment

In order to further validate the effectiveness of our algorithm, we used a power system transmission fault dataset [38], with four 11 kV generators located at each end of the line. Transformers were included to facilitate various fault scenarios at the midpoint of the line. The dataset comprises line currents (Ia, Ib, Ic) and line voltages (Va, Vb, Vc) measured under normal operation and multiple fault conditions, including single-phase-to-ground (LG), line-to-line (LL), double-line-to-ground (LLG), and three-phase (LLL) faults. Following the same approach used for the TE data, we divided the dataset into segments of 40 samples each, resulting in a total of 4825 samples across five data types. Among these, 3825 samples were used for training, 500 for validation, and 500 for testing. Because the dataset was relatively small, the batch size was set to 64, the learning rate to 0.0001, and the training ran for 100 epochs. We applied early stopping after 30 consecutive epochs without improvement on the validation set, and the remaining training parameters were consistent with previous experiments. The results in Table 6 show a 100% recognition rate across all fault categories, reaffirming the algorithm’s effectiveness and underscoring its broad applicability in both the chemical and power industries.

## 5. Conclusions

This paper proposed a ViT-based global and local feature fusion algorithm for high-dimensional time-series fault diagnosis. The algorithm effectively combines the global features obtained by the learnable classification vector in traditional ViT with the local features extracted by the Transformer encoder, applying image recognition methods to the diagnosis of dynamic and complex industrial process faults. Compared to existing SOTA algorithms, whether based on CNN improvements or ViT improvements, our proposed algorithm demonstrates superior performance. In the TE dataset for fault diagnosis, the proposed algorithm achieves an average F1-score of 98.37% and an average recall of 98.38% across all data types, including normal states, surpassing the advanced algorithms we referenced. This further proves the advancement and effectiveness of our model. Directly applying ViT to time-series data may involve several potential risks, such as insufficient modeling capability for temporal dependencies, inappropriate preprocessing methods, a lack of interpretability and trustworthiness, as well as challenges in generalization and robustness. Additionally, ViT requires significant computational resources, which could limit its efficiency. To fully realize the potential of ViT in time-series analysis, more systematic and in-depth research is needed in areas such as algorithmic improvement, model evaluation, and interpretability analysis. At the same time, attention should be given to the latest technological advancements and practical experiences to continuously optimize and refine relevant methods. This will ultimately contribute to the development of smarter, more reliable, and efficient fault diagnosis tools for industrial applications.

## Figures and Tables

**Table 1:** Number of data segments for different segment lengths.

Segment Length (n)	5	10	20	40
Train	4,598,160	4,549,860	4,453,260	4,260,060
Validation	199,920	197,820	193,620	185,220
Test	199,920	197,820	193,620	185,220

**Table 2:** Performance comparison between GLF-ViT and other algorithms.

	Ours			IPO-VIT			Target-Transformer			LGS-CNN			CNN-LSTM			ANN			FDA		
Class	R	P	F1	R	P	F1	R	P	F1	R	P	F1	R	P	F1	R	P	F1	R	P	F1
	(%)			(%)			(%)			(%)			(%)			(%)			(%)		
0	100.00	100.00	100.00	90.20	92.00	91.09	93.00	78.95	85.40	NA	NA	NA	NA	NA	NA	NA	NA	NA	NA	NA	NA
1	100.00	100.00	100.00	100.00	100.00	100.00	99.75	98.22	98.98	100.00	100.00	100.00	100.00	100.00	100.00	100.00	99.50	99.70	99.00	100.00	100.00
2	100.00	100.00	100.00	100.00	97.50	98.73	98.44	99.12	98.78	100.00	99.90	99.50	100.00	100.00	100.00	100.00	100.00	100.00	100.00	100.00	100.00
3	99.35	90.95	94.97	84.44	95.00	89.41	99.38	77.98	87.39	94.00	91.30	92.60	96.50	81.10	88.10	80.50	69.70	74.70	21.00	7.00	11.00
4	100.00	100.00	100.00	98.77	100.00	99.38	99.63	99.31	99.47	99.50	100.00	99.70	100.00	99.00	99.50	98.50	96.60	97.50	18.00	27.00	21.00
5	100.00	100.00	100.00	100.00	100.00	100.00	91.88	90.18	91.02	100.00	100.00	100.00	100.00	100.00	100.00	99.50	98.50	99.00	29.00	41.00	34.00
6	100.00	100.00	100.00	100.00	100.00	100.00	98.21	100.00	99.10	100.00	100.00	100.00	100.00	100.00	100.00	100.00	100.00	100.00	100.00	100.00	100.00
7	100.00	100.00	100.00	100.00	100.00	100.00	99.94	100.00	99.97	100.00	100.00	100.00	100.00	100.00	100.00	100.00	100.00	100.00	89.00	100.00	94.00
8	100.00	99.91	99.95	100.00	98.75	99.37	95.56	99.03	97.26	98.50	100.00	99.20	98.50	98.50	98.50	97.50	87.40	92.20	56.00	58.00	56.00
9	79.24	97.15	87.29	85.07	71.25	77.55	68.69	85.93	76.35	89.00	80.20	84.40	72.00	74.60	73.30	29.50	32.40	30.90	13.00	10.00	11.00
10	99.84	99.99	99.91	100.00	93.75	96.77	97.69	99.87	98.77	95.80	98.00	98.30	92.50	96.90	94.60	88.50	80.80	84.50	32.00	28.00	30.00
11	100.00	100.00	100.00	100.00	98.75	99.37	98.06	99.18	98.62	100.00	99.50	99.80	99.00	100.00	99.50	82.00	96.50	88.60	26.00	18.00	21.00
12	99.90	99.68	99.79	100.00	100.00	100.00	97.06	99.17	98.10	100.00	97.60	98.80	87.00	87.00	87.00	85.00	92.40	88.50	56.00	42.00	48.00
13	99.34	99.83	99.59	92.68	95.00	93.83	96.13	99.35	97.71	98.50	100.00	99.20	92.00	99.50	95.60	94.50	100.00	97.20	68.00	52.00	59.00
14	100.00	100.00	100.00	100.00	100.00	100.00	98.75	98.69	98.72	100.00	100.00	100.00	100.00	100.00	100.00	99.50	100.00	99.70	16.00	17.00	16.00
15	96.24	83.94	89.67	65.52	71.25	68.26	34.06	53.12	41.51	86.50	94.00	90.10	74.00	76.70	75.30	53.00	42.60	47.20	10.00	9.00	9.00
16	100.00	100.00	100.00	78.95	75.00	76.92	52.69	42.02	46.76	98.50	99.50	99.00	96.00	97.00	96.50	69.50	69.80	69.70	15.00	17.00	16.00
17	99.16	99.81	99.48	98.72	96.25	97.47	94.75	99.87	97.24	99.00	100.00	99.50	99.00	98.50	98.80	98.00	99.00	98.50	100.00	94.00	97.00
18	95.14	97.81	96.45	88.24	93.75	90.91	94.25	98.50	96.33	95.50	100.00	97.70	87.50	87.10	87.30	90.50	99.50	94.80	100.00	94.00	97.00
19	99.74	100.00	99.87	100.00	98.75	99.37	98.69	93.54	96.05	100.00	99.50	99.80	100.00	98.00	99.00	65.50	77.10	70.80	17.00	31.00	22.00
20	98.03	99.51	98.76	96.34	98.75	97.53	94.25	100.00	97.04	98.50	99.50	99.00	97.50	99.50	98.50	95.50	96.40	96.00	100.00	95.00	98.00
Ave.	**98.38**	**98.50**	**98.37**	94.23	94.08	94.09	90.52	91.05	90.50	97.80	97.90	97.80	94.60	94.70	94.60	86.30	86.90	86.50	53.00	52.00	52.00

R represents recall, P represents precision, F1 represents F1 score, NA represents not available.

**Table 3:** Recall and precision for different segment lengths.

	Seq-5		Seq-10		Seq-20		Seq-40	
Class	R	P	R	P	R	P	R	P
	(%)		(%)		(%)		(%)	
0	100.00	100.00	100.00	100.00	100.00	100.00	100.00	100.00
1	99.97	100.00	100.00	99.79	100.00	100.00	100.00	100.00
2	99.63	99.94	99.99	99.95	100.00	100.00	100.00	100.00
3	81.75	56.19	92.00	68.13	95.24	84.96	98.00	90.97
4	99.96	99.43	100.00	99.85	100.00	99.95	100.00	100.00
5	100.00	99.91	100.00	99.97	100.00	100.00	100.00	100.00
6	100.00	100.00	100.00	100.00	100.00	100.00	100.00	100.00
7	100.00	100.00	100.00	99.98	100.00	100.00	100.00	99.79
8	97.39	99.77	98.35	99.86	99.86	100.00	100.00	99.97
9	30.50	66.18	44.16	79.92	65.70	89.77	76.84	95.75
10	95.68	99.18	97.62	99.64	98.23	99.66	99.88	99.93
11	98.31	99.45	99.51	100.00	99.90	100.00	100.00	100.00
12	97.79	99.89	99.70	100.00	99.93	100.00	99.94	99.38
13	93.12	99.26	94.88	98.35	96.76	99.00	99.31	99.81
14	100.00	100.00	100.00	100.00	100.00	100.00	100.00	100.00
15	70.65	49.43	82.89	61.95	92.39	72.69	96.34	81.47
16	97.41	99.84	99.04	99.97	99.71	99.87	99.97	100.00
17	93.53	99.78	94.45	98.68	97.46	99.87	99.21	99.93
18	93.03	97.80	91.52	96.43	93.63	97.25	94.50	98.38
19	99.81	100.00	99.93	99.97	99.69	99.92	99.71	100.00
20	93.38	99.00	92.77	99.06	96.75	98.63	98.32	99.80
Ave.	92.47	93.67	94.61	95.31	96.87	97.22	**98.19**	**98.34**

**Table 4:** Performance comparison with different head of feature fusion and layer configurations.

Head	Layer	R (%)	P (%)	F1 (%)
16	3	98.19	98.34	98.18
	4	98.23	98.41	98.21
	5	97.90	98.03	97.88
32	3	98.14	98.32	98.11
	4	**98.38**	**98.50**	**98.37**
	5	98.05	98.20	98.04
64	4	98.24	98.41	98.22

**Table 5:** F1 comparison of four models (a), (b), (c), and (d) across different classes.

Model	a (%)	b (%)	c (%)	d (%)
Fault 3	94.46	94.59	94.12	**94.97**
Fault 9	84.94	84.18	84.83	**87.29**
Fault 15	89.03	89.16	88.05	**89.67**
Ave.	98.22	98.23	98.10	**98.37**

**Table 6:** Performance of fault detection in power system transmission lines.

Class	Normal	LG	LL	LLG	LLL
P	100%	100%	100%	100%	100%
R	100%	100%	100%	100%	100%
F1	100%	100%	100%	100%	100%

## Data Availability

No new data were created or analyzed in this study. Data sharing is not applicable to this article.

## References

[1] Jiang Y., Yin S., Kaynak O. (2020). Performance supervised plant-wide process monitoring in industry 4.0: A roadmap. IEEE Open J. Ind. Electron. Soc..

[2] Ge Z. (2017). Review on data-driven modeling and monitoring for plant-wide industrial processes. Chemom. Intell. Lab. Syst..

[3] López-Estrada F.-R., Astorga-Zaragoza C.-M., Theilliol D., Ponsart J.-C., Valencia-Palomo G., Torres L. (2017). Observer synthesis for a class of Takagi–Sugeno descriptor system with unmeasurable premise variable. Application to fault diagnosis. Int. J. Syst. Sci..

[4] Lee J., Yoo C., Lee I. (2004). Statistical process monitoring with independent component analysis. J. Process Contr..

[5] Ardali N., Zarghami R., Gharebagh R. (2024). Optimized data driven fault detection and diagnosis in chemical processes. Comput. Chem. Eng..

[6] Shahzad F., Huang Z., Memon W. (2022). Process monitoring using kernel PCA and kernel density estimation-based SSGLR method for nonlinear fault detection. Appl. Sci..

[7] Shi Q., Zhang H. (2021). Fault diagnosis of an autonomous vehicle with an improved SVM algorithm subject to unbalanced datasets. IEEE Trans. Ind. Electron..

[8] Li C., Sanchez R.V., Zurita G., Cerrada M., Cabrera D., Vásquez R.E. (2016). Gearbox fault diagnosis based on deep random forest fusion of acoustic and vibratory signals. Mech. Syst. Signal Process..

[9] Barrera-Llanga K., Burriel-Valencia J., Sapena-Bano A., Martinez-Roman J. (2025). Fault detection in induction machines using learning models and Fourier spectrum image analysis. Sensors.

[10] Zhang J., Zhang Q., Qin X., Sun Y. (2024). Robust fault diagnosis of quayside container crane gearbox based on 2D image representation in frequency domain and CNN. Struct. Health Monit..

[11] Yan J., Liu T., Ye X., Jing X., Dai Y. (2021). Rotating machinery fault diagnosis based on a novel lightweight convolutional neural network. PLoS ONE.

[12] Song Q., Jiang P. (2022). A multi-scale convolutional neural network based fault diagnosis model for complex chemical processes. Process Saf. Environ..

[13] Xu M., Gao J., Zhang Z., Wang H. (2022). Bearing-fault diagnosis with signal-to-rgb image mapping and multichannel multiscale convolutional neural network. Entropy.

[14] Xiao B., Zhang Y., Zhou C., Ou J., Huang G. (2024). A noise-robust CNN architecture with global attention and gated convolutional Kernels for bearing fault detection. Meas. Sci. Technol..

[15] Dong Z., Zhao D., Cui L. (2024). An intelligent bearing fault diagnosis framework: One-dimensional improved self-attention-enhanced CNN and empirical wavelet transform. Nonlinear Dynam..

[16] Debasish J., Jayant P., Sudheendra H., Satish N. (2022). CNN and Convolutional Autoencoder (CAE) based real-time sensor fault detection, localization, and correction. Mech. Syst. Signal Process..

[17] Saif S., Wahaibi A., Abiola S., Lu Q. (2023). Improving convolutional neural networks for fault diagnosis in chemical processes by incorporating global correlations. Comput. Chem. Eng..

[18] Khan M.A., Choo J., Kim Y. (2020). Intelligent fault detection using raw vibration signals via dilated convolutional neural networks. J. Supercomput..

[19] Ildar L., Mark L., Ilya M. (2021). Fault detection in Tennessee Eastman process with temporal deep learning models. J. Ind. Inf. Integr..

[20] Huang T., Zhang Q., Tao X., Zhao S., Lu X. (2022). A novel fault diagnosis method based on CNN and LSTM and its application in fault diagnosis for complex systems. Artif. Intell. Rev..

[21] Meng X., Tan H., Yan P., Zheng Q., Chen G., Jiang J. (2024). A GNSS/INS Integrated Navigation Compensation Method Based on CNN–GRU + IRAKF Hybrid Model During GNSS Outages. IEEE Trans. Instrum. Meas..

[22] Li M., Peng P., Sun H., Wang M., Wang H. (2023). An order-invariant and interpretable dilated convolution neural network for chemical process fault detection and diagnosis. IEEE Trans. Autom. Sci. Eng..

[23] Li Y., Liu Z., Jia Z., Zhao W., Wang K., Qin X. (2024). Fault Diagnosis Strategy for Flight Control Rudder Circuit Based on SHAP Interpretable Analysis Optimization Transformer with Attention Mechanism. IEEE Trans. Instrum. Meas..

[24] Li Z., Liu F., Yang W., Peng S., Zhou J. (2022). A survey of convolutional neural networks: Analysis, applications, and prospects. IEEE Trans. Neur. Net. Lear. Syst..

[25] Vaswani A. Attention is all you need. Proceedings of the Advances in Neural Information Processing Systems 30: Annual Conference on Neural Information Processing Systems 2017.

[26] Dosovitskiy A. An image is worth 16 × 16 words: Transformers for image recognition at scale. Proceedings of the International Conference on Learning Representations.

[27] Zhu Q., Qian Y., Zhang N., He Y., Xu Y. (2023). Multi-scale Transformer-CNN domain adaptation network for complex processes fault diagnosis. J. Process Control.

[28] Wei C., Han H., Wu Z., Xia Y., Ji Z. (2024). Transformer-Based Multiscale Reconstruction Network for Defect Detection of Infrared Images. IEEE Trans. Instrum. Meas..

[29] Liu S., Yu H., Liao C., Lin J. Pyraformer: Low-complexity pyramidal attention for long-range time series modeling and forecasting. Proceedings of the Tenth International Conference on Learning Representations (ICLR 2022).

[30] Zhou H., Zhang S., Peng S., Zhang J., Li J., Xiong H., Zhang W. Informer: Beyond efficient transformer for long sequence time-series forecasting. Proceedings of the AAAI Conference on Artificial Intelligence.

[31] Zhang Y., Wu R., Dascalu S., Harris F. (2024). Multi-scale transformer pyramid networks for multivariate time series forecasting. IEEE Access.

[32] Wang J., Ma S., An Y., Dong R. (2024). A Comparative Study of Vision Transformer and Convolutional Neural Network Models in Geological Fault Detection. IEEE Access.

[33] Kang Y., Chen G., Wang H., Shen J., Wei X. (2023). Fault anomaly detection method of aero-engine rolling bearing based on distillation learning. ISA Trans..

[34] Zhou K., Tong Y., Li X., Huang H., Song K., Chen X. (2023). Exploring global attention mechanism on fault detection and diagnosis for complex engineering processes. Process Saf. Environ..

[35] Downs J., Vogel E. (1993). A plant-wide industrial process control problem. Comput. Chem. Eng..

[36] Amsel R., Tran B., Maia R. (2017). Additional tennessee eastman process simulation data for anomaly detection evaluation. Harv. Dataverse.

[37] Wei Z., Xu J., Li Z., Dang Y., Dai Y. (2022). A novel deep learning model based on target transformer for fault diagnosis of chemical process. Process Saf. Environ..

[38] Jamil M., Sharma S.K., Singh R. (2015). Fault detection and classification in electrical power transmission system using artificial neural network. SpringerPlus.

